# Driving Laboratory Standardization of Bacterial Culture and Antimicrobial Susceptibility Testing in Veterinary Clinical Microbiology in Europe and Beyond

**DOI:** 10.1128/JCM.02572-20

**Published:** 2021-05-19

**Authors:** Dorina Timofte, Els M. Broens, Luca Guardabassi, Constanca Pomba, Fergus Allerton, John Ikonomopoulos, Gudrun Overesch, Peter Damborg

**Affiliations:** a Institute of Infection, Veterinary and Ecological Sciences, School of Veterinary Science, Department of Veterinary Anatomy, Physiology and Pathology, University of Liverpool, Leahurst, United Kingdom; b Department of Biomolecular Health Sciences, Faculty of Veterinary Medicine, Utrecht University, Utrecht, the Netherlands; c Department of Veterinary and Animal Sciences, Faculty of Health and Medical Sciences, University of Copenhagen, Frederiksberg, Denmark; d Department of Pathobiology and Population Sciences, The Royal Veterinary College, North Mymms, United Kingdom; e Antibiotic Resistance Laboratory, Center for Interdisciplinary Research in Animal Health (CIISA), Faculty of Veterinary Medicine, University of Lisbon, Lisbon, Portugal; f Willows Veterinary Center and Referral Service, Shirley, Solihull, United Kingdom; g School of Animal Biosciences, Department of Animal Science, Laboratory of Anatomy and Physiology of Farm Animals, Agricultural University of Athens, Athens, Greece; h Institute of Veterinary Bacteriology, University of Bern, Bern, Switzerland; Emory University

**Keywords:** standardization, veterinary microbiology

## Abstract

Globally, antimicrobial resistance is one of the most important public health challenges in which the clinical microbiology laboratory plays a critical role by providing guidance for antimicrobial treatment. Despite the recognition of its importance, there is still a real need for the standardized training of clinical microbiologists and harmonization of diagnostic procedures. This is particularly true for veterinary clinical microbiology, where additional challenges exist when microbiologists are trying to fulfill a professional role very similar to that of their colleagues working in human microbiology laboratories. The specific points that need addressing to improve the outputs of veterinary microbiology laboratories discussed here include (i) harmonization of methodologies used by veterinary laboratories for antimicrobial susceptibility testing (AST); (ii) specific guidelines for interpretation and reporting of AST results for animal pathogens; (iii) guidelines for detection of antimicrobial resistance mechanisms in animal isolates; (iv) standardization of diagnostic procedures for animal clinical specimens; and (v) the need to train more veterinary clinical microbiology specialists. However, there is now a plan to address these issues, led by the European Network for Optimization of Veterinary Antimicrobial Treatment (ENOVAT), which is bringing together experts in veterinary microbiology, pharmacology, epidemiology, and antimicrobial stewardship from Europe and wider afield. ENOVAT is aiming to work with project partners toward standardization and harmonization of laboratory methodologies and optimization of veterinary antimicrobial treatment. Ultimately, the project may provide a mechanism for standardization and harmonization of veterinary clinical microbiology methodologies that could then be used as a template for implementation at a wider international level.

## INTRODUCTION

Antimicrobial resistance (AMR) is a global multifactorial issue that endangers the ability to treat bacterial infections and hinders the implementation of important medical advances (i.e., complex surgeries and chemotherapy) in both human and veterinary medicine. The emergence of AMR has highlighted the key role that clinical microbiology laboratories play in driving antimicrobial stewardship and appropriate antimicrobial use (1).

The underuse or suboptimal use of microbiological culture and antimicrobial susceptibility testing (AST) and overreliance on empirical antimicrobial therapy can exacerbate AMR in both human and veterinary settings; therefore, to overcome these obstacles, a closer partnership between diagnostic laboratories and clinicians is required for successful antimicrobial stewardship (1, 2). In addition, there have been calls for standardized the training of clinical microbiologists and a better understanding of the professional identity of clinical microbiologists, in line with the recognition received by other specialties (3, 4). If calls for greater professional recognition are warranted in human clinical microbiology, where the field is already seen as an integral element of antimicrobial stewardship, a similar need exists for both closer laboratory-clinic collaboration and improved recognition of the role of clinical microbiologists in veterinary settings. To facilitate these needs, standardized training of veterinary clinical microbiologists, a better recognition of the clinical microbiologist’s role in patient care, and harmonization of professional standards are needed in veterinary clinical microbiology. In addition, several major challenges exist for veterinary microbiology laboratories, which we discuss here.

## HARMONIZING METHODOLOGIES OF AST IN VETERINARY LABORATORIES

Although international AST standards for microbiology laboratories exist and are largely applicable to veterinary settings (https://www.iso.org/standard/70464.html), their implementation is dependent on local factors. Furthermore, currently there is no worldwide consensus for the use of a common methodology in veterinary laboratories. When performing culture and AST, veterinary laboratories generally follow methodologies developed for processing human clinical isolates. In that regard, laboratories adhere to the European Committee on Antimicrobial Susceptibility Testing (EUCAST) or the American Clinical and Laboratory Standards Institute (CLSI) guidelines, or, less commonly, guidelines issued by various national committees. This approach serves the immediate needs of clinicians, and the data can be useful for detecting shifts in local antimicrobial susceptibility patterns. However, the use of multiple standards is a major limitation when comparing susceptibility data between laboratories or countries, thereby compromising global AMR surveillance in animal pathogens. Hence, the early detection of emergent resistant pathogens or meaningful comparison of resistance rates within or between countries is hampered, as shown in a study comparing antimicrobial susceptibility data in canine urinary tract infection isolates from across Europe (5). Similarly, human studies have shown that the usefulness of AMR surveillance is often jeopardized by variability in laboratory procedures or noncompliance with international reporting standards (6). In addition, the quality management guidance provided by CLSI for monitoring antimicrobial resistance trends using cumulative susceptibility data provided by human epidemiologic studies (7) also needs to be followed in veterinary surveillance programs.

## LACK OF SPECIFIC GUIDELINES FOR INTERPRETATION AND REPORTING OF AST RESULTS FOR ANIMAL PATHOGENS

Although veterinary antimicrobial susceptibility testing subcommittees have been established within both the CLSI (-VAST) and EUCAST (VetCAST), there is still a shortage of animal-, infection-, and pathogen-specific clinical breakpoints (CBPs) for antimicrobial drugs used in veterinary medicine. Both subcommittees are actively developing more clinical breakpoints for veterinary antimicrobial agents; however, this is a slow process due to the complexity of the tasks for various pathogen-antimicrobial combinations in different infections and animal hosts. In the meantime, the lack of specific interpretative criteria for animal pathogens represents a great difficulty for laboratory staff. Thus, developing best practice guidelines for interpreting and reporting AST results for animal pathogens for which CBPs are not yet available must be regarded as a priority for the veterinary profession.

## LACK OF GUIDELINES FOR DETECTION OF AMR MECHANISMS IN CLINICAL COMPANION ANIMAL ISOLATES

AMR is widespread in companion and livestock animals (8, 9), and accurate detection and identification of resistant organisms is paramount for infection control and preventing zoonotic transmission. Although harmonization of methods and interpretative criteria for monitoring AMR in zoonotic and commensal bacteria from healthy food-producing animals has been established through the EU-Commission Decision 2013/652/EU (https://www.eumonitor.eu/9353000/1/j9vvik7m1c3gyxp/vk0vn25n5e9o), AMR surveillance in companion animals, primarily cats, dogs, and horses, has not been included. Veterinary laboratories, which actively perform AMR surveillance, often follow either the CLSI (10) or EUCAST procedures (https://www.eucast.org/fileadmin/src/media/PDFs/EUCAST_files/Resistance_mechanisms/EUCAST_detection_of_resistance_mechanisms_170711.pdf) for specific detection of resistance mechanisms; however, these are not entirely applicable for veterinary clinical isolates. For instance, consensus on detection methods for methicillin resistance in important animal pathogens, such as methicillin-resistant Staphylococcus pseudintermedius (MRSP) or *S. schleiferi* (MRSS), is still lacking (10, 11). In addition, detection of these and other multidrug-resistant (MDR) organisms emerging in companion animals (e.g., carbapenem-resistant Escherichia coli and Acinetobacter baumannii [12, 13]) is often restricted to specialized research laboratories, raising the question of whether many AMR issues remain undetected. All of this points to a clear need for guidance for veterinary laboratories on screening and reporting policies, including when to refer emerging MDR organisms to specialist laboratories.

## STANDARDIZATION OF DIAGNOSTIC PROCEDURES FOR ANIMAL CLINICAL SPECIMENS

The absence of specific guidelines and methodologies for processing animal clinical specimens for microbiology testing is a well-recognized and serious challenge to the profession (14). Consequently, there is an urgent need for the standardization of the diagnostic process from sample collection, processing, pathogen identification, selection of isolates for AST, and reporting in veterinary laboratories across all veterinary service providers. Such a lack of specific guidelines for common procedures in veterinary laboratories has multiple implications that influence the appropriate diagnosis and clinical management of infections, directly impacting antimicrobial stewardship. Thus, AMR surveillance programs may become ineffectual, inappropriate therapeutic interventions, and significant zoonoses may go undetected. A comprehensive set of recommended clinical microbiology procedures, covering all stages of microbiological investigations, is necessary to ensure common standards across microbiology laboratories processing veterinary specimens. These should include guidelines for (i) clinical specimen collection and laboratory management specific to the clinical condition/animal species, (ii) specimen-specific culture, (iii) organism isolation and identification, (iv) the selection of relevant bacterial pathogens for AST, and (v) the interpretation and reporting of culture and susceptibility results. A widely available resource for such protocols, similar to what is available for human microbiology laboratories in the United Kingdom (Standards for Microbiology Investigations [UK SMIs]; https://www.gov.uk/guidance/uk-standards-for-microbiology-investigations-smi-quality-and-consistency-in-clinical-laboratories), should be created through a similar consultation process involving all partners and organizations active in this field. Ideally, these laboratory procedures should be standardized at a European level and made available to all veterinary microbiology laboratories. In addition, a new framework for Microbiology Investigation Criteria for Reporting Objectively (MICRO), to ensure accurate and comparable microbiology laboratory results are produced among human laboratories, was recently published and could be adopted by veterinary laboratories (15).

Although the points highlighted here are long-held goals, there is now a plan for action that is being led by the European Network for Optimization of Veterinary Antimicrobial Treatment (ENOVAT). ENOVAT is an EU COST Action project bringing together experts in veterinary microbiology, pharmacology, epidemiology, and antimicrobial stewardship throughout Europe and wider afield via collaborations with Near Neighbor Countries and International Partner Countries. Among other important objectives (https://enovat.eu/about/), ENOVAT is aiming to use online surveys to critically review the current methodologies and interpretive criteria used by veterinary microbiology diagnostic laboratories and identify gaps and challenges of microbiological diagnostic procedures. The survey outcome will provide an invaluable data source that can be used to draw a roadmap outlining how ENOVAT can work with project partners toward standardization and harmonization of veterinary microbiology methodologies.

## THE ROLE OF VETERINARY CLINICAL MICROBIOLOGISTS IN THE CONTEXT OF EMERGING MOLECULAR TECHNOLOGIES

Similar to humans, animal infections are often caused by opportunistic pathogens residing in the commensal bacterial population, making interpretation of culture results and pathogen selection for AST challenging (16). The optimization of this process requires the expertise of a clinical microbiologist, ideally with a veterinary background, to guide the laboratory technical staff, to give advice at all analytical stages, and to facilitate the dialogue between the laboratory and clinicians. Such dialogue is increasingly important due to the advent and uptake of new laboratory diagnostic technologies. For example, matrix-assisted laser desorption ionization–time of flight (MALDI-TOF) mass spectrometry has been increasingly adopted as the gold standard for bacterial and fungal identification in veterinary microbiology laboratories (17–19). MALDI-TOF has revolutionized clinical microbiology by introducing an easy-to-perform, rapid, low-cost method of identification; however, veterinary microbiologists also need to be aware of the new challenges arising, as the low cost of testing per isolate can lead to more isolates being identified to species level than in the pre-MALDI-TOF era. To reduce the risks of “overidentification,” a very careful process of “clinical microbiology reasoning” needs to be undertaken by the bench microbiologist to ensure that only isolates that are clinically relevant are selected for AST (20, 21). Although the occurrence of technical errors in laboratory testing is reduced by following quality control programs, interpretation of culture results should integrate multiple clinical and laboratory factors to identify and pursue clinically significant bacterial isolates. The wealth of knowledge built up in human clinical microbiology studies shows that the underestimation of the value of this process can lead to testing and reporting of organisms not associated with infection and, hence, contribute to inappropriate or ineffective antimicrobial therapy (22).

Furthermore, new molecular tools aiming to improve diagnostic quality or speed up result turnaround time have emerged in clinical microbiology. These molecular diagnostic technologies are designed to detect a single or multiple pathogen(s) (bacterial, viral, or fungal) associated with clinical syndromes. These molecular tools include point-of-care tests (POCTs), gene-based resistance detection platforms, single or multiplex PCR assays, immune-chromatographic tests, peptide nucleic acid fluorescent *in situ* hybridization (FISH) technologies, loop-mediated isothermal assays (LAMP), mass spectrometry, and next-generation sequencing (NGS) (21, 23). POCTs, also known as rapid diagnostic tests or near patient tests, are used in both human and animal settings; these are designed to be used outside the laboratory and to generate results in under an hour, allowing timely interventions. A recent study that sought to identify POCTs currently available for diagnosing animal disease in developing countries has found that many POCTs target a small number of key zoonotic animal diseases, while few exist for other important animal diseases (24). This study also highlighted that the lack of validation regulations for veterinary POCTs has allowed tests that have been improperly validated to enter the market, presenting challenges for customers and undermining their true potential in disease control (24). Multiplex PCR assays have the advantage of simultaneously detecting multiple bacterial, viral, and/or fungal pathogens likely to be associated with a particular clinical syndrome (e.g., respiratory, gastrointestinal [GI], sepsis, or central nervous system [CNS] infections); however, the disadvantage is that novel unsuspected pathogens may be missed (21). These multiplex detection platforms have gained a place in human and veterinary clinical practice, as they support timely detection and clinical management decisions, but they have also introduced challenges in the clinical microbiology laboratory. These include cost-value analysis, integration of molecular platforms in the laboratory workflow, and the need for experienced specialists for results interpretation and monitoring results accuracy (21). Not last, these molecular advances include NGS and bioinformatics, which are increasingly used for high-resolution typing of pathogens or plasmids during hospital outbreaks, detection of genes associated with antimicrobial resistance, or pathogenicity, although they are more commonly undertaken as part of research investigations (25). The role of whole-genome sequencing (WGS) in predicting AST was reviewed by Ellington et al., who concluded that, currently, for most bacterial species there is insufficient evidence to support the use of WGS-inferred AST to guide clinical decision-making (26). Furthermore, direct pathogen detection in clinical specimens (metagenomics NGS) via Nanopore MinION sequencing is gaining popularity due to the advantages provided by its novel features (compact portable device providing real-time sequencing and analysis), allowing easier integration in the microbiology laboratory workflow (27). However, the transition of NGS from research to the clinical human and veterinary clinical laboratory setting seems to be a distant prospect due to its complexity and the need for expert input, especially bioinformatics knowledge required for interpretation of results, as well as validation and quality assurance (28). The issues around availability and integration of molecular diagnostics in the human and veterinary routine microbiology laboratory workflow are even more profound in developing countries due to poor infrastructures, financial inequities, and lack of training. In addition, there is a lack of effective AMR surveillance networks and diagnostic capacity in both human and animal populations in developing countries, leading to an increased use of broad-spectrum antimicrobials by health professionals (29).

As technical advances continue to emerge in clinical microbiology, careful integration of what is technically possible with what is clinically relevant will require regular appropriate training of staff to keep pace with the developments in the field (23, 27). This highlights the importance of veterinary clinical microbiology training and specialization, which has a longstanding history in America, where the American College of Veterinary Microbiology was formed in 1968 (https://www.acvm.us/about-acvm/). In Europe, the formation of the European College of Veterinary Microbiology (ECVM) became a reality in 2016 (https://ebvs.eu/colleges/ECVM). In addition, the Study Group of Veterinary Microbiology (ESGVM), established within the European Society for Clinical Microbiology and Infectious Diseases, also promotes the need for training and specialization in veterinary microbiology in Europe (https://www.escmid.org/research_projects/study_groups/study_groups_o_z/veterinary_microbiology/). Furthermore, the European Association for Veterinary Diagnosticians (EAVLD; https://www.eavld.org/eavld/) provides a platform for networking and communication among veterinary laboratories.

Ultimately, the increasing threat from AMR and zoonotic emerging infectious diseases underlies the need to improve and integrate veterinary microbiology services with public health services worldwide to provide the backbone of a global One Health approach. Ensuring that veterinary microbiology laboratories have the technical facilities and the expertise of veterinary microbiology specialists provides the necessary infrastructure to change and adapt to new challenges, such as the one represented by the SARS-COV-2 pandemic. This major public health issue has created unprecedented pressure on global health services and provided an opportunity for veterinary microbiology services to rise to the challenge and show their adaptability by joining the global effort of controlling the pandemic through PCR testing when it was most needed (30).

## SUMMARY

Within the ENOVAT project, we are developing united complementary approaches in the veterinary microbiology profession to help achieve the long-held goals of harmonization of AST methods and standardization of diagnostic procedures across veterinary microbiology laboratories in Europe and beyond. We are also lobbying for more training of clinical veterinary microbiologists to enable the rollout of high-quality diagnostic and treatment protocols for animals. This would ensure the implementation of common strategies and a level playing field across all laboratories, which will positively reduce the AMR burden and ultimately improve animal and public health. The outcomes may well bring benefits to veterinary diagnosticians worldwide.

## References

[1] Morency-Potvin P, Schwartz DN, Weinstein RA. 2017. Antimicrobial stewardship: how the microbiology laboratory can right the ship. Clin Microbiol Rev 30:381–407. doi:10.1128/CMR.00066-16. 27974411 PMC5217798

[2] Guardabassi L, Apley M, Olsen JE, Toutain P-L, Weese S. 2018. Optimization of antimicrobial treatment to minimize resistance selection. Microbiol Spectr 6:e0018-17. doi:10.1128/microbiolspec.ARBA-0018-2017. PMC11633575 29932044

[3] Humphreys H, Nagy E, Kahlmeter G, Ruijs GJHM. 2010. The need for European professional standards and the challenges facing clinical microbiology. Eur J Clin Microbiol Infect Dis 29:617–621. doi:10.1007/s10096-010-0906-2. 20333424 PMC7088261

[4] Yusuf E, Ong DSY, Martin-Quiros A, Skevaki C, Cortez J, Dedić K, Maraolo AE, Dušek D, Maver PJ, Sanguinetti M, Tacconelli E, Trainee Association of the European Society of Clinical Microbiology and Infectious Diseases (ESCMID). 2017. A large survey among European trainees in clinical microbiology and infectious disease on training systems and training adequacy: identifying the gaps and suggesting improvements. Eur J Clin Microbiol Infect Dis 36:233–242. doi:10.1007/s10096-016-2791-9. 27704297 PMC5253151

[5] Marques C, Gama LT, Belas A, Bergström K, Beurlet S, Briend-Marchal A, Broens EM, Costa M, Criel D, Damborg P, van Dijk MAM, van Dongen AM, Dorsch R, Espada CM, Gerber B, Kritsepi-Konstantinou M, Loncaric I, Mion D, Misic D, Movilla R, Overesch G, Perreten V, Roura X, Steenbergen J, Timofte D, Wolf G, Zanoni RG, Schmitt S, Guardabassi L, Pomba C. 2016. European multicenter study on antimicrobial resistance in bacteria isolated from companion animal urinary tract infections. BMC Vet Res 12:213. doi:10.1186/s12917-016-0840-3. 27658466 PMC5034465

[6] Williams PCM, Isaacs D, Berkley JA. 2018. Antimicrobial resistance among children in sub-Saharan Africa. Lancet Infect Dis 18:e33–e44. doi:10.1016/S1473-3099(17)30467-X. 29033034 PMC5805911

[7] Hindler JF, Stelling J. 2007. Analysis and presentation of cumulative antibiograms: a new consensus guideline from the Clinical and Laboratory Standards Institute. Clin Infect Dis 44:867–873. doi:10.1086/511864. 17304462

[8] Joosten P, Ceccarelli D, Odent E, Sarrazin S, Graveland H, Van Gompel L, Battisti A, Caprioli A, Franco A, Wagenaar JA, Mevius D, Dewulf J. 2020. Antimicrobial usage and resistance in companion animals: a cross-sectional study in three European countries. Antibiotics 9:87. doi:10.3390/antibiotics9020087. PMC7175148 32079072

[9] Jakobsen L, Kurbasic A, Skjøt-Rasmussen L, Ejrnaes K, Porsbo LJ, Pedersen K, Jensen LB, Emborg HD, Agersø Y, Olsen KE, Aarestrup FM, Frimodt-Møller N, Hammerum AM. 2010. Escherichia coli isolates from broiler chicken meat, broiler chickens, pork, and pigs share phylogroups and antimicrobial resistance with community-dwelling humans and patients with urinary tract infection. Foodborne Pathog Dis 7:537–547. doi:10.1089/fpd.2009.0409. 20039794

[10] Saab ME, Muckle CA, Stryhn H, McClure JT. 2018. Comparison of culture methodology for the detection of methicillin-resistant Staphylococcus pseudintermedius in clinical specimens collected from dogs. J Vet Diagn Invest 30:93–98. doi:10.1177/1040638717729396. 29020886 PMC6504161

[11] Skov R, Varga A, Matuschek E, Åhman J, Bemis D, Bengtsson B, Sunde M, Humphries R, Westblade L, Guardabassi L, Kahlmeter G. 2020. EUCAST disk diffusion criteria for the detection of mecA-mediated β-lactam resistance in Staphylococcus pseudintermedius: oxacillin versus cefoxitin. Clin Microbiol Infect 26:122.e1–122.e6. doi:10.1016/j.cmi.2019.05.002. 31108230

[12] Reynolds ME, Phan HTT, George S, Hubbard ATM, Stoesser N, Maciuca IE, Crook DW, Timofte D. 2019. Occurrence and characterization of Escherichia coli ST410 co-harbouring blaNDM-5, blaCMY-42 and blaTEM-190 in a dog from the UK. J Antimicrob Chemother 74:1207–1211. doi:10.1093/jac/dkz017. 30753576

[13] Pomba C, Endimiani A, Rossano A, Saial D, Couto N, Perreten V. 2014. First report of OXA-23-mediated carbapenem resistance in sequence type 2 multidrug-resistant Acinetobacter baumannii associated with urinary tract infection in a cat. Antimicrob Agents Chemother 58:1267–1268. doi:10.1128/AAC.02527-13. 24295971 PMC3910809

[14] Guardabassi L, Damborg P, Stamm I, Kopp PA, Broens EM, Toutain PL, ESCMID Study Group for Veterinary Microbiology. 2017. Diagnostic microbiology in veterinary dermatology: present and future. Vet Dermatol 28:146. doi:10.1111/vde.12414. 28133869

[15] Turner P, Fox-Lewis A, Shrestha P, Dance DAB, Wangrangsimakul T, Cusack T-P, Ling CL, Hopkins J, Roberts T, Limmathurotsakul D, Cooper BS, Dunachie S, Moore CE, Dolecek C, van Doorn HR, Guerin PJ, Day NPJ, Ashley EA. 2019. Microbiology Investigation Criteria for Reporting Objectively (MICRO): a framework for the reporting and interpretation of clinical microbiology data. BMC Med 17:70. doi:10.1186/s12916-019-1301-1. 30922309 PMC6440102

[16] Price LB, Hungate BA, Koch BJ, Davis GS, Liu CM. 2017. Colonizing opportunistic pathogens (COPs): the beasts in all of us. PLoS Pathog 13:e1006369. doi:10.1371/journal.ppat.1006369. 28796836 PMC5552013

[17] Becker P, Normand A-C, Vanantwerpen G, Vanrobaeys M, Haesendonck R, Vercammen F, Stubbe D, Piarroux R, Hendrickx M. 2019. Identification of fungal isolates by MALDI-TOF mass spectrometry in veterinary practice: validation of a web application. J Vet Diagn Invest 31:471–474. doi:10.1177/1040638719835577. 30943879 PMC6838695

[18] Randall LP, Lemma F, Koylass M, Rogers J, Ayling RD, Worth D, Klita M, Steventon A, Line K, Wragg P, Muchowski J, Kostrzewa M, Whatmore AM. 2015. Evaluation of MALDI-ToF as a method for the identification of bacteria in the veterinary diagnostic laboratory. Res Vet Sci 101:42–49. doi:10.1016/j.rvsc.2015.05.018. 26267088

[19] Spergser J, Hess C, Loncaric I, Ramírez AS. 2019. Matrix-assisted laser desorption ionization–time of flight mass spectrometry is a superior diagnostic tool for the identification and differentiation of mycoplasmas isolated from animals. J Clin Microbiol 57:e00316-19. doi:10.1128/JCM.00316-19. 31217275 PMC6711924

[20] Wilson ML. 1997. Clinically relevant, cost-effective clinical microbiology. Strategies to decrease unnecessary testing. Am J Clin Pathol 107:154–167. doi:10.1093/ajcp/107.2.154. 9024064

[21] Messacar K, Parker SK, Todd JK, Dominguez SR. 2017. Implementation of rapid molecular infectious disease diagnostics: the role of diagnostic and antimicrobial stewardship. J Clin Microbiol 55:715–723. doi:10.1128/JCM.02264-16. 28031432 PMC5328439

[22] Baron EJ. 2011. The role of the clinical microbiology laboratory in the diagnosis of selected infectious processes. J Clin Microbiol 49:S25–S25. doi:10.1128/JCM.00842-11.

[23] Buchan BW, Ledeboer NA. 2014. Emerging technologies for the clinical microbiology laboratory. Clin Microbiol Rev 27:783–822. doi:10.1128/CMR.00003-14. 25278575 PMC4187641

[24] Hobbs EC, Colling A, Gurung RB, Allen J. 15 October 2020. The potential of diagnostic point-of-care tests (POCTs) for infectious and zoonotic animal diseases in developing countries: technical, regulatory and sociocultural considerations. Transbound Emerg Dis doi:10.1111/tbed.13880. PMC8359337 33058533

[25] Rossen JWA, Friedrich AW, Moran-Gilad J, ESCMID Study Group for Genomic and Molecular Diagnostics (ESGMD). 2018. Practical issues in implementing whole-genome-sequencing in routine diagnostic microbiology. Clin Microbiol Infect 24:355–360. doi:10.1016/j.cmi.2017.11.001. 29117578

[26] Ellington MJ, Ekelund O, Aarestrup FM, Canton R, Doumith M, Giske C, Grundman H, Hasman H, Holden MTG, Hopkins KL, Iredell J, Kahlmeter G, Köser CU, MacGowan A, Mevius D, Mulvey M, Naas T, Peto T, Rolain JM, Samuelsen Ø, Woodford N. 2017. The role of whole genome sequencing in antimicrobial susceptibility testing of bacteria: report from the EUCAST Subcommittee. Clin Microbiol Infect 23:2–22. doi:10.1016/j.cmi.2016.11.012. 27890457

[27] Petersen LM, Martin IW, Moschetti WE, Kershaw CM, Tsongalis GJ. 2019. Third-generation sequencing in the clinical laboratory: exploring the advantages and challenges of nanopore sequencing. J Clin Microbiol 58:e01315-19. doi:10.1128/JCM.01315-19. 31619531 PMC6935936

[28] Gargis AS, Kalman L, Lubin IM. 2016. Assuring the quality of next-generation sequencing in clinical microbiology and public health laboratories. J Clin Microbiol 54:2857–2865. doi:10.1128/JCM.00949-16. 27510831 PMC5121372

[29] Vernet G, Mary C, Altmann DM, Doumbo O, Morpeth S, Bhutta ZA, Klugman KP. 2014. Surveillance for antimicrobial drug resistance in under-resourced countries. Emerg Infect Dis 20:434–441. doi:10.3201/EID2003.121157. 24564906 PMC3944851

[30] Stokol T, McAloose D, Terio KA, Salguero FJ. 2020. Severe acute respiratory syndrome-coronavirus-2 (SARS-CoV-2): a perspective through the lens of the veterinary diagnostic laboratory. Front Vet Sci 7:576267. doi:10.3389/fvets.2020.576267. 33088836 PMC7544814

